# A moderately higher time-in-range threshold improves the prognosis of type 2 diabetes patients complicated with COVID-19

**DOI:** 10.3389/fendo.2024.1353838

**Published:** 2024-07-02

**Authors:** Riping Cong, Jianbo Zhang, Lujia Xu, Yujian Zhang, Hao Wang, Jing Wang, Wei Wang, Yingli Diao, Haijiao Liu, Jing Zhang, Kuanxiao Tang

**Affiliations:** ^1^ Department of General Practice, Qilu Hospital of Shandong University, Jinan, Shandong, China; ^2^ Department of Emergency and Chest Pain Center, Qilu Hospital of Shandong University, Jinan, Shandong, China; ^3^ Department of Pulmonary and Critical Care Medicine, Qilu Hospital of Shandong University, Jinan, Shandong, China; ^4^ Department of Internal Medicine, Jinan Hospital, Jinan, Shandong, China; ^5^ Department of Endocrinology, Lanling County Traditional Chinese Medicine Hospital, Linyi, Shandong, China

**Keywords:** type 2 diabetes, COVID-19, time-in-range, continuous glucose monitoring, glucose variability

## Abstract

**Objective:**

After fully lifting coronavirus disease 2019 (COVID-19) pandemic control measures in mainland China in 12/2022, the incidence of COVID-19 has increased markedly, making it difficult to meet the general time-in-range (TIR) requirement. We investigated a more clinically practical TIR threshold and examined its association with the prognosis of COVID-19 patients with type 2 diabetes(T2D).

**Research design and methods:**

63 T2D patients complicated with COVID-19 were evaluated. Patients were divided into favorable outcome group and adverse outcome group according to whether achieving composite endpoint (a >20-day length of stay, intensive care unit admission, mechanical ventilation use, or death). TIR, the time-below-range (TBR) and the time-above-range (TAR) were calculated from intermittently scanned continuous glucose monitoring. Logistic regression analysis and other statistical methods were used to analyze the correlation between glucose variability and prognosis to establish the appropriate reference range of TIR.

**Results:**

TIR with thresholds of 80 to 190 mg/dL was significantly associated with favorable outcomes. An increase of 1% in TIR is connected with a reduction of 3.70% in the risk of adverse outcomes. The Youden index was highest when the TIR was 54.73%, and the sensitivity and specificity were 58.30% and 77.80%, respectively. After accounting for confounding variables, our analysis revealed that threshold target ranges (TARs) ranging from 200 mg/dL to 230 mg/dL significantly augmented the likelihood of adverse outcomes.

**Conclusion:**

The TIR threshold of 80 to 190 mg/dL has a comparatively high predictive value of the prognosis of COVID-19. TIR >54.73% was associated with a decreased risk of adverse outcomes. These findings provide clinically critical insights into possible avenues to improve outcomes for COVID-19 patients with T2D.

## 1 Introduction

The Chinese Center for Disease Control and Prevention had reported that since the pandemic control measures of coronavirus disease 2019 (COVID-19) were fully lifted in mainland China in 12/2022, the peak number of COVID-19 nucleic acid-positive cases had reached 6.94 million, admissions to hospitals had reached a peak of 1.625 million, of which the highest number of severe cases had reached 128 thousand, and the cumulative number of deaths had reached 4273 by January 2023.

Diabetes has already become the second most common comorbidity of COVID-19 due to the coinciding of two global pandemics (1, 2). A meta-analysis including 7 studies with 1,576 patients showed the prevalence of diabetes of approximately 9.7% (95% CI: 7.2–12.2%) (3). Another meta-analysis was a comprehensive systematic search including data from 76,993 patients (4). According to this study, the prevalence of diabetes was estimated to be 7.87% (95% CI: 6.57–9.28%). Poor glycemic control increased the risk of mortality, morbidity, and secondary infections (5, 6).

These associations between diabetes and worse outcomes in COVID-19 patients were incontrovertible, as blood glucose fluctuation was not conducive to the improvement of disease, and inflammation caused by hyperglycemia led to increased mortality (7, 8). However, excessively tight glycemic control may increase the risk of hypoglycemia, which also increased mortality (9).The impact of COVID-19 on the patients and the use of glucocorticoids and nutritional support during the treatment increased blood glucose fluctuations, which had adverse effects on the prognosis (10). The UK Diabetes guidelines recommend a blood glucose target of 110 to 180mg/dL for diabetes patients with COVID-19, and a blood glucose level of less than 220mg/dL for patients with hypoglycemia and high risk factors (including the elderly, patients with low body weight, patients with severe COVID-19 and/or renal impairment) (11). American Diabetes Association guidelines recommend targeting blood glucose < 180 mg/dL in critically ill patients (12). Clinicians face a significant challenge in improving outcomes for individuals with COVID-19 and type 2 diabetes(T2D) due to uncertainty surrounding the optimal degree of glycemic management and its potential impact on treatment benefits and risks. The definition of optimal blood glucose control remains controversial (13). The wide application of hormonal and nutritional support treatment has led to significant fluctuations in blood glucose levels in clinical practice, making it challenging to maintain the general range. Consequently, our study aimed to analyze glycemic profiles using intermittently scanned continuous glucose monitoring (isCGM) to determine a more clinically practical threshold for TIR and investigate its correlation with prognosis.

## 2 Materials and methods

In our observational study, data of patients admitted to Qilu Hospital of Shandong University (public tertiary care) from Dec 2022 to Apr 2023 were analyzed. The patients all had moderate or severe cases and were diagnosed according to the guidelines issued by the World Health Organization (WHO) (14), meeting at least the following criteria: positive COVID-19 RNA PCR and characteristic imaging manifestations of novel coronavirus pneumonia. Additionally, eligible patients were those with T2D that had been diagnosed before admission or with newly diagnosed T2D after admission. All patients met the diagnostic criteria of T2D: typical diabetes symptoms plus random blood glucose ≥11.1 mmol/L or plus fasting plasma glucose(FPG)≥7.0 mmol/L or OGTT 2h blood glucose≥11.1 mmol/L. Exclusion criteria included patients who were intubated on admission and those younger than 18 years of age. The study was approved by the Ethics Committees of Qilu Hospital of Shandong University (KYLL-202307–047). Trial Registration: clinicaltrials.gov Identifier: NCT06156137 (Registered November 24, 2023).

Patient information that we collected through electronic medical records include gender, age, vital signs, symptoms on admission, duration of diabetes, comorbidities, FPG, hemoglobin A_1c_ (HbA_1c_), alanine aminotransferase (ALT), aspartate aminotransferase (AST), total protein (TP), total cholesterol (TC), triglycerides (TG), serum creatinine, uric acid, estimated glomerular filtration rate (eGFR), inflammatory biomarkers, brain natriuretic peptide (BNP), CK-MB and medication, including oral hypoglycemic agent (OHA), insulin, anticoagulant drugs and glucocorticoids. CGM was initiated on admission. Diabetic meals were ordered for all patients during hospitalization.

All patients were equipped with isCGM sensors (FreeStyle Libre Flash glucose monitoring system; Abbott Diabetes Care Ltd, UK) on admission, and the nurse retrieved the probe when the patient was discharged or when the composite endpoint was reached. The routine protocol for glucose monitoring during hospitalization was fixed at four swipes daily (fasting, premeal and bedtime). In addition, scans can be performed when the patient encounters symptoms of hypoglycemia or any other discomfort. Measures of glycemic variability, such as time-in-range (TIR), time-below-range (TBR) and time-above-range (TAR), mean sensor glucose level and coefficient of variation (CV) of glucose levels, were calculated from isCGM records. TIR was defined as the percentage of time within the following ranges: 70–180 mg/dL, 80–190 mg/dL, 90–200 mg/dL, 100–210 mg/dL, 110–220 mg/dL, and 120–230 mg/dL.

A composite adverse outcome included a hospital stay of more than 20 days, admission to the intensive care unit, the need for mechanical ventilation, and death.

### 2.1 Statistical analysis

All data were analyzed using the SPSS software v.25(IBM Corporation, Armonk, NY). The normal distribution of continuous variables was checked by the Shapiro-Wilk test. Nonnormally distributed variables are presented as the median (IQR), and the Mann-Whitney U test and Kruskal-Wallis ANOVA were used for comparisons between groups. Categorical variables were expressed as numbers (percentages) and were compared using the χ^2^ test or Fisher’s exact test. To identify the covariates for inclusion in the multivariate analysis, a univariate logistic regression was initially performed. Candidate covariates were selected based on a significance level of *P* < 0.05 in the univariate analysis. Subsequently, multivariable-adjusted logistic regression models were employed to evaluate the association between TIR using isCGM and composite adverse outcomes. All analyses were adjusted for age, sex, CK-MB, symptoms on admission, LDH, use of OHA and anticoagulant. A receiver-operating characteristic (ROC) curve was constructed with TIR as the independent variable and prognosis as the dependent variable, and the diagnostic value of TIR was assessed based on the area under the curve (AUC). The optimal cutoff value was determined using the Youden index. All statistical tests were two-sided, and a significance level of *P* < 0.05 was considered statistically significant. Odds ratios (ORs) with 95% CIs are presented.

## 3 Results

### 3.1 Clinical characteristics of patients with COVID-19 and T2D upon admission

**Figure 1:**
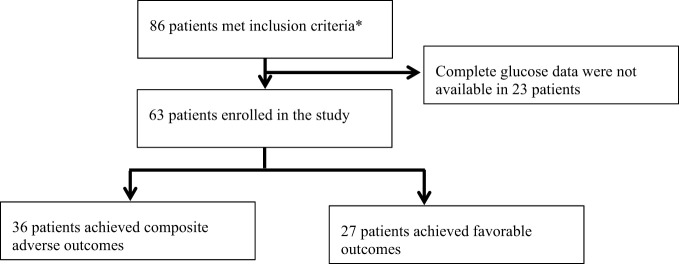
Trial profile. *Meeting the following criteria: 1. Inpatients; 2. Patients diagnosed with T2D; 3. Patients receiving CGM during hospitalization; 4. Positive COVID-19 RNA PCR and characteristic imaging manifestations of novel coronavirus pneumonia.

This study included a total of 63 patients who met the inclusion criteria (**Figure 1**). Among them, the mean age was 71.59 ± 12.24 years, including 42.90% female and 57.10% male. 27 of the 63 patients experienced composite adverse outcomes. The characteristics of these patients are presented in **Table 1**. Patients with adverse composite outcome had obvious cardiac damage on admission, and the myocardial injury markers LDH (280[234,342.75] U/L vs. 242[194.25,286] U/L) and CK-MB (2.20 [1.30,4.20] ng/mL vs. 1.50[0.78,2.10] ng/mL) were significantly increased (*P*<0.05). Additionally, patients in the adverse outcome group appeared to be older(75 vs. 72 years), accompanied by comorbidities(hypertension:70% vs. 67%; coronary heart disease: 70% vs. 56%) and higher levels of CRP(67.29[20.84,127.00] mg/L vs. 41.72 [7.51,95.99]mg/L), D-dimmer(1.76 [0.96,2.85] μg/mL vs. 0.97[0.56,1.81]μg/mL) on admission but there was no significant difference between the two groups.

**Table 1:** Characteristics and isCGM data of patients with COVID-19 and T2D.

Parameters	Presence of the composite adverse outcome	
	No (n = 36)	Yes (n = 27)
Clinical Characteristics on Admission		
Age (years)	72 (62, 82)	75 (65,80)
Male gender	21 (58.30)	15 (55.56)
Heart rate (bpm)	80 (71,72)	84 (80,99)
Respiratory rate (bpm)	18 (18,20)	20 (18,21)
SBP (mmHg)	130 (118,142)	133 (120,147)
DBP (mmHg)	76 (68,80)	77 (70,81)
Fatigue	18 (50)	13 (48)
Dyspnea	20 (56)	14 (52)
Comorbidities on Admission		
Hypertension	24 (67)	19 (70)
Coronary heart disease	20 (56)	12 (70)
Chronic renal diseases	8 (22)	5 (19)
Laboratory Examination on Admission		
Leukocyte count (10^9^/L)	7.37 (5.68,10.05)	7.54 (5.84,10.19)
Neutrophil count (10^9^/L)	5.63 (3.54,8.51)	6.60 (4.60,8.05)
Lymphocyte count (10^9^/L)	1.27 (0.73,1.54)	0.84 (0.57,1.30)
C-reactive protein (mg/L)	41.72 (7.51,95.99)	67.29 (20.84,127.00)
Procalcitonin level (ng/mL)	0.12 (0.07,0.32)	0.28 (0.14,0.94)
ALT (U/L)	18 (10,25)	16 (13,30)
AST (U/L)	20 (15,28)	26 (16,33)
Creatinine (μmol/L)	68.50 (53.25,103)	91 (55,144)
eGFR (mL/min/1.73 m^2^)	86.69 (57.76,100.65)	55.40 (36.83,98.58)
CK (U/L)	44.00 (25.50,67)	65.00 (26.5,121.25)
CK-MB (ng/mL)	1.50 (0.78,2.10)^*^	2.20 (1.30,4.20)^*^
LDH (U/L)	242(194.25,286)^*^	280(234,342.75)^*^
Triglycerides (mmol/L)	1.35 (0.96,1.64)	1.43 (0.94,1.9)
LDL cholesterol (mmol/L)	2.25 (1.67,3.28)	1.95 (1.55,2.51)
HDL cholesterol (mmol/L)	1.08 (0.83,1.33)^*^	0.94 (0.72,1.09)^*^
D-dimer (μg/mL)	0.97 (0.56,1.81)	1.76 (0.96,2.85)
FPG (mg/dL)	7.53 (6.54,16.15)	13.07 (9.43,16.46)
HbA_1c_ (%)	7.6 (6.8,9.33)	8.15 (6.78,10.13)
Sensor glucose (mg/dL)	177.84 (153.70,217.95)^*^	222.84 (183.33,283.49)^*^
Coefficient of variation (%)	32.95 (28.95,37.15)	34.35 (27.23,37.93)
Treatment		
Antibiotic therapy	35 (97)	25 (93)
Glucocorticoids	24 (67)	23 (85)
Anticoagulant Therapy	14 (39)^*^	19 (70)^*^
Non-insulin Hypoglycemic Agents	25 (69)^*^	12 (44)^*^
insulin Hypoglycemic Agents	21 (58)	21 (78)

Data were presented as n (%) or median (IQR). *P < 0.05

ALT, alanine aminotransferase; AST, aspartate aminotransferase; eGFR, estimated glomerular filtration rate; CK, creatine kinase; CK-MB, creatine kinase-myocardial isoenzyme.

### 3.2 Clinical treatment of patients with COVID-19 and T2D

The treatment of hospitalized patients with T2D and COVID-19 mainly includes anti-inflammatory therapy, hypoglycemic therapeutics and other nutritional support therapy. More than 70% of the 63 patients were treated with glucocorticoids therapy, 75% of the patients were treated with nutritional support, 52.4% of the patients were treated with anticoagulant therapy and 66.7% of the patients were treated with insulin. Compared with the improved discharge group, more patients in the poor outcome group used anticoagulant therapy (70% vs. 39%, *P*<0.05). There was no significant difference in the use of antibiotics (93% vs. 97%), glucocorticoids (85% vs. 67%) and insulin (78% vs. 58%) between the two groups (*P*>0.05).

### 3.3 Comparison of TIR between the adverse and favorable outcome groups

**Figure 2:**
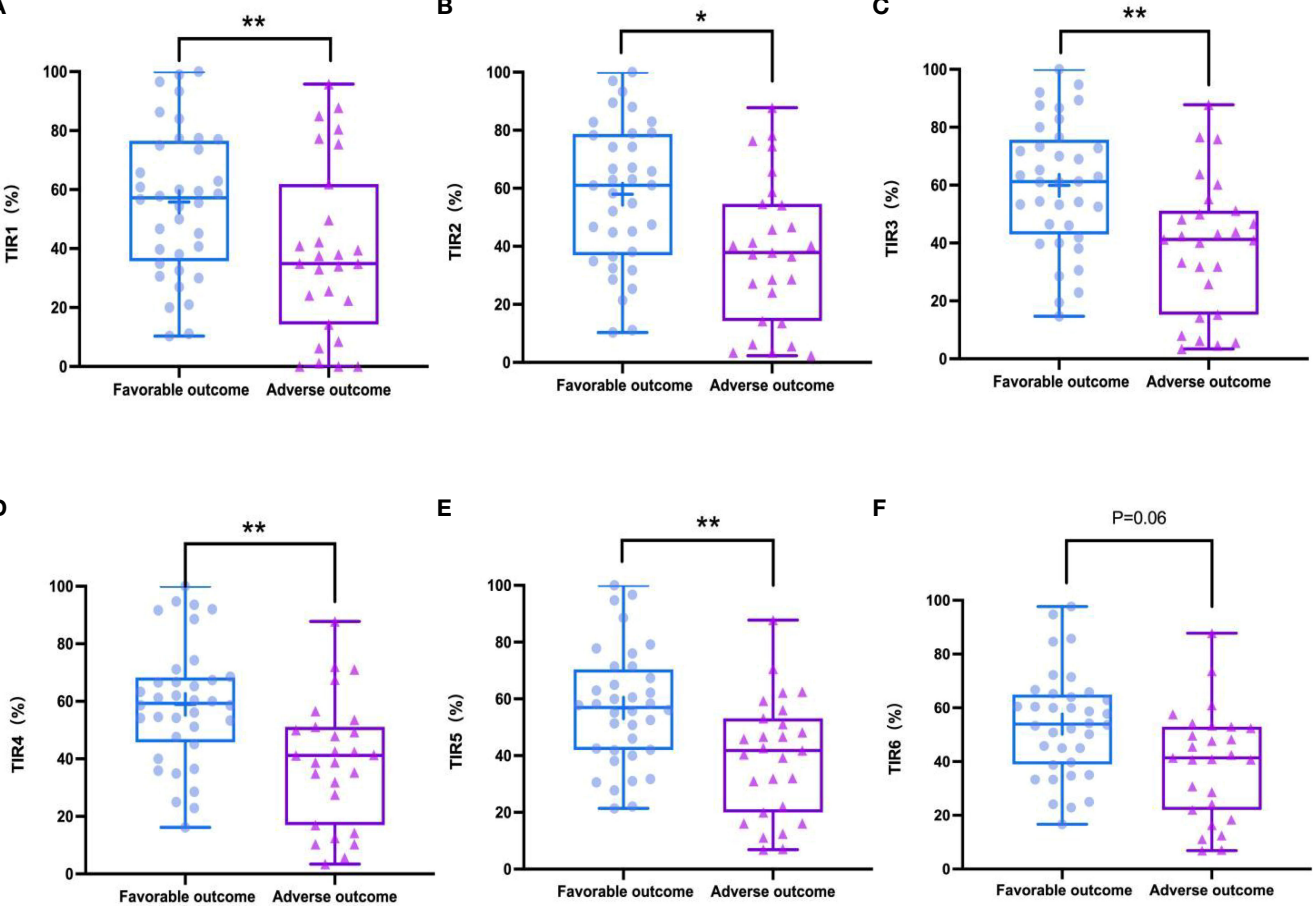
TIRs in favorable outcomes and adverse outcomes groups during hospitalization. Adverse outcomes showed significantly lower TIR1 (70-180 mg/dL) than favorable outcomes **(A)**, TIR2 (80-190 mg/dL) **(B)**, TIR3 (90-200 mg/dL) **(C)**, TIR4 (100-210 mg/dL) **(D)**, TIR5 (110-220 mg/dL) **(E)** but TIR6 (120-230 mg/dL) had a weakly negative correlation with the outcomes **(F)**. *P < 0.05, **P < 0.01.

**Figure 3:**
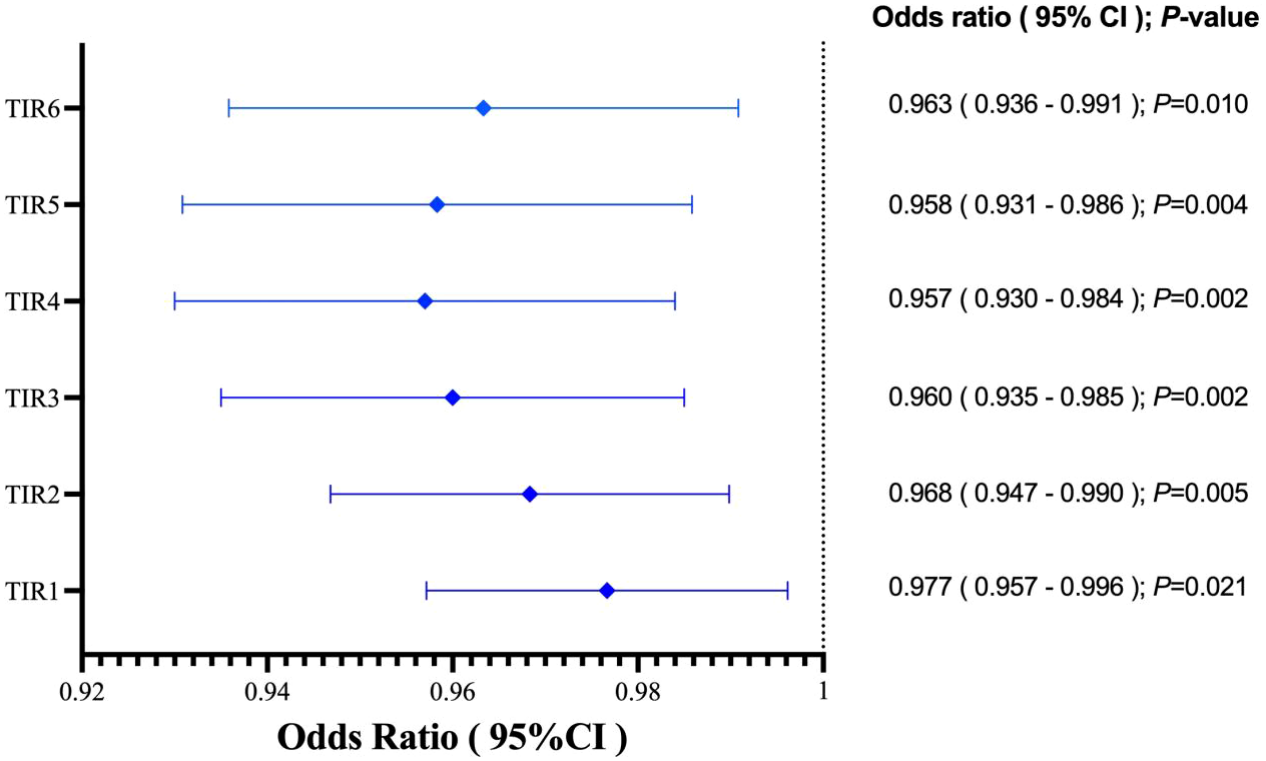
TIRs variables was associated with decreased risk of the adverse outcome. TIR1:70-180 mg/dL; TIR2:80-190 mg/dL; TIR3:90-200 mg/dL; TIR4:100-210 mg/dL; TIR5:110-220 mg/dL; TIR6:120-230 mg/dL.

In the study, the mean TIR (70–180mg/dl) of patients was 48.57%, the mean sensor glucose level was 203.57 (162.7–235.88) mg/dl, and the mean CV was 33.29% (27.88 – 37.62). The proportion of patients with TIR (70–180mg/dL)>70% during hospitalization was 26.9%. Patients with composite adverse outcomes exhibited significantly lower TIR values compared to those with favorable outcomes (*P* < 0.05) (**Figure 2**). Univariate and multivariable logistic regression models were used to analyze data from all 63 patients. Univariate regression analysis showed that TIR variables (0.977 [0.957–0.996], 0.968 [0.947–0.990], 0.960 [0.935–0.985], 0.957 [0.930–0.984], 0.958 [0.931–0.986], 0.963 [0.936–0.991]) were associated with a decreased risk of the composite outcome (**Figure 3**). Univariate logistic regression analysis of composite outcomes is shown in **Table 2**. After adjustment for multiple covariates (age, sex, CK-MB, symptoms on admission, LDH, use of OHA and anticoagulant), TIRs (0.975 [0.948–1.002], 0.963 [0.932–0.995], 0.951 [0.916–0.988], 0.950 [0.914–0.987], 0.960 [0.926–0.995], 0.967 [0.934–1.001]) exhibited a significant association with reduced odds of composite adverse outcomes (**Table 3**). Thus, a TIR of 80–190 mg/dL was significantly associated with favorable outcomes.

**Table 2:** Univariate logistic regression analysis of composite outcomes of COVID-19.

	Odds ratios (95% confidence interval)	P
Clinical Characteristics on Admission		
Age (years)	1.015(0.973,1.058)	0.489
Male gender	1.120(0.409,3.068)	0.826
Heart rate (bpm)	1.014(0.850,1.045)	0.345
Respiratory rate (bpm)	0.998(0.944,1.033)	0.584
SBP(mmHg)	1.009(0.988,1.031)	0.410
DBP(mmHg)	1.005(0.969,1.041)	0.803
Fatigue	0.929(0.342,2.520)	0.929
Dyspnea	0.862(0.317,2.344)	0.770
Gastrointestinal symptoms	2.125(0.33,13.704)	0.428
Comorbidities on Admission		
Hypertension	1.187(0.404,3.490)	0.755
Coronary heart disease	0.640(0.234,1.747)	0.640
Chronic renal diseases	0.795(0.228,2.774)	0.720
Laboratory Examination on Admission		
Leukocyte count (10^9^/L)	1.073(0.958,1.203)	0.224
Neutrophil count (10^9^/L)	1.107(0.973,1.260)	0.123
Lymphocyte count (10^9^/L)	0.617(0.257,1.480)	0.279
C-reactive protein (mg/L)	1.006(0.998,1.015)	0.122
Procalcitonin level (ng/mL)	0.950(0.831,1.085)	0.447
ALT (U/L)	1.004(0.962,1.049)	0.844
AST (U/L)	1.038(0.985,1.095)	0.160
Creatinine (μmol/L)	0.999(0.996,1.003)	0.801
eGFR (mL/min/1.73 m^2^)	0.991(0.976,1.006)	0.244
CK (U/L)	1.004(0.998,1.010)	0.175
CK-MB (ng/ml)	1.543(1.070,2.224)	0.020
LDH(U/L)	1.009(1.001,1.017)	0.021
Triglycerides (mmol/L)	1.299(0.775,2.180)	0.321
LDL cholesterol (mmol/L)	0.593(0.322,1.094)	0.094
HDL cholesterol (mmol/L)	0.184(0.032,1.040)	0.055
D-dimer (μg/mL)	1.129(0.966,1.319)	0.128
FPG (mg/dL)	1.000(0.965,1.037)	0.986
HbA_1c_ (%)	1.129(0.900,1.578)	0.221
Sensor glucose (mg/dL)	1.010(1.001,1.019)	0.031
Coefficient of variation (%)	1.007(0.945,1.073)	0.831
Treatment		
Antibiotic therapy	0.357(0.031,4.158)	0.411
Glucocorticoids	0.348(0.098,1.236)	0.103
Anticoagulant Therapy	3.732(1.288,10.812)	0.015
Non-insulin Hypoglycemic Agents	0.352(0.125,0.995)	0.049
Insulin Hypoglycemic Agents	2.500(0.813,7.689)	0.110

**Table 3:** Multivariate analysis for predicting composite adverse outcomes by glycemic metrics derived from isCGM.

	Odds ratios (95% confidence interval)
Sensor glucose levels (mg/dL)	
TIR	
TIR1(70-180)	0.975 (0.948-1.002)
TIR2(80-190)	0.963 (0.932-0.995)
TIR3(90-200)	0.951 (0.916-0.988)
TIR4(100-210)	0.950 (0.914-0.987)
TIR5(110-220)	0.960 (0.926-0.995)
TIR6(120-230)	0.967 (0.934-1.001)

Data are adjusted for age, sex, CK-MB, symptoms on admission, LDH, Use of OHA and anticoagulant.

### 3.4 TIR predicted the prognosis of T2D patients with COVID-19

**Figure 4:**
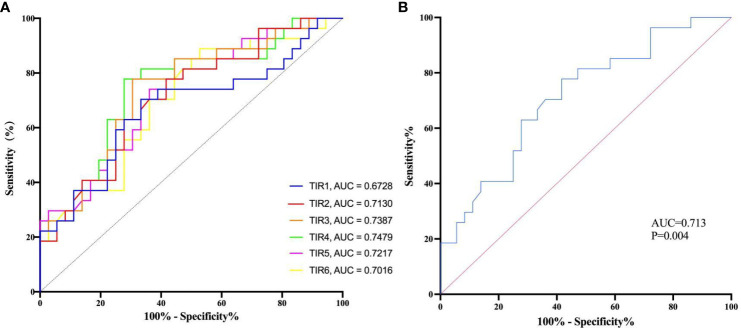
The diagnostic value of TIR was evaluated by the receiver operating characteristic (ROC) analysis. **(A)** The receiver operating characteristic (ROC) analysis was used to evaluate the diagnostic value of TIRs. TIR1:70-180 mg/dL; TIR2:80-190 mg/dL; TIR3:90-200 mg/dL; TIR4:100-210 mg/dL; TIR5:110-220 mg/dL; TIR6:120-230 mg/dL; AUC, area under curve. **(B)** The receiver operating characteristic (ROC) analysis was used to evaluate the diagnostic value of TIR of 80-190 mg/dL and estimate the optimal cutoff value.

The multivariate logistic regression analysis revealed that the TIRs of 80–190, 90–200, and 100–210 mg/dL remained as independent predictors of composite adverse outcomes even after adjusting the multiple covariates. ROC analysis was employed to evaluate the prognostic value of TIR for COVID-19 patients with T2D. The test variables were defined as TIRs within the ranges of 80–190, 90–200, and 100–210mg/dL while the state variable was represented by composite adverse outcomes in patients (**Figure 4A**). The area under the ROC curve was 0.713 (95% CI: 0.585–0.841, *P* = 0 .004), 0.739 (95% CI: 0.614–0.863, *P* = 0 .0013), and 0.748 (95% CI: 0.624–0.872, *P* < 0 .001). The area under the curve is maximized when TIR exhibits high predictive value for COVID-19 patient prognosis. Although the TIR (100–210 mg/dL) had the largest area under the ROC curve, it was not significantly different from the other two ROC curves. This does not indicate that the TIR (100–210 mg/dL) has higher prognostic value than the TIR (80–190 mg/dL) and the TIR (90–200 mg/dL). In this study, the average TIR (80–190mg/dl) of patients with adverse composite outcome was significantly lower than that of the favorable outcome group (38.29 ± 24.94% vs. 57.94 ± 24.42%, *P*<0.05), while TAR was significantly higher (55.59 ± 31.35% vs. 39.82 ± 25.47%, *P*<0.05). Therefore, glycemic control between 80 and 190mg/dl can improve the prognosis. In all patients, the TIR of 80–190 mg/dL corresponds to 54.73% and maximizes the Youden index, with a sensitivity and specificity of 58.3% and 77.8%, respectively (**Figure 4B**).

## 4 Discussion

Data from this cross-sectional study showed that optimal glycemic control during hospitalization was associated with a lower risk of severe illness and death in patients with COVID-19. After adjusting for covariates, maintaining TIR within the thresholds of 80 to 190 mg/dL significantly relates to favorable outcomes.

In our study, the patient population was divided into two cohorts based on the occurrence of composite adverse events. The proportion of severe COVID-19 cases at admission was higher in the population with composite adverse events than in the second cohort (63% vs. 33.3%, *P* = 0.002). Although patients with composite adverse outcomes were more likely to be male and older than 65 years with comorbidities and higher levels of inflammatory, endothelial, and coagulopathy markers on admission, there was no significant difference between the two groups. Patients achieving composite adverse outcomes had significantly higher CK-MB and LDH levels on admission. When analyzing TIR as a factor influencing outcome, all of the above confounding variables were adjusted for to reach the following conclusion: TIR values with thresholds of 80 to 190 mg/dL were significantly associated with a lower risk of the composite adverse outcomes.

Previous studies have shown that variability is a potential risk predictor of death and other complications (4, 15). The presence of COVID-19 has been shown to play a significant role in impairing blood glucose control within the range of 70–150 mg/dL (13). A study of 548 patients with COVID-19 and T2D has confirmed that the parameters such as mean glucose, peak glucose, and the magnitude of glycemic fluctuations in the early stage of hospitalization are significantly correlated with adverse outcomes, and are closely related to increased hospitalization expenses, prolonged hospitalization time, and increased risk of all-cause death (16). A small-sample study (17) suggested that maintaining TIR (70–160 mg/dL) >70% could improve outcomes. In clinical practice, we found that only 15.87% of patients achieved that target, and the average TIR in our study was 39.36% during the pandemic. Inpatient medication (corticosteroids) and enteral and parenteral nutrition contribute to hyperglycemia (18). The widespread use of glucocorticoids caused patients to experience wide fluctuations in blood glucose levels, which may have more adverse effects than sustained hyperglycemia. In our study, more than 70% of the patients were received glucocorticoids therapy, and 75% were treated with enteral or parenteral nutrition, which resulted in a high mean sensor glucose level [203.57 mg/dL (162.7–235.88)] and a wide CV of glucose values [33.29% (27.88 to 37.62)]. This also explained why the TIR threshold of COVID-19 patients with T2D was higher.

Moreover, the elevation of cortisol levels resulting from COVID-19 infection, stress, and similar factors can contribute to excessive hepatic gluconeogenesis, impaired glucose utilization, and insulin deficiency (19–21). There is a suggested direct impact of SARS-CoV-2 on pancreatic β-cell function and survival, exacerbating rapid and severe metabolic deterioration in individuals with preexisting diabetes (22, 23). Angiotensin-converting enzyme 2 (ACE 2) potentially serves as a crucial molecular link between COVID-19 severity and insulin resistance (23–25). Our findings supported this hypothesis, as the patients who achieved the composite adverse outcomes had a significantly lower TIR (80–190 mg/dL) and a higher TAR >190 mg/dL. Furthermore, they used a higher maximum insulin dose during hospitalization [34(18–47) vs. 19(0–40), *P* = 0.046]. In this study, we found that poor glycemic control was associated with a worse outcome that included a higher need for medical intervention, hospitalization, and mortality. The insights gained here provide direct suggestions for the clinical management of T2D during the COVID-19 pandemic.

Excessive glycemic control leading to severe hypoglycemia has been associated with increased mortality rates (9). The international consensus on TIR (26) indicated that although evidence regarding TIR for older or high-risk individuals is limited, several studies have demonstrated an elevated risk for hypoglycemia. Therefore, they reduced the TIR target from 70% to 50%. In our study, the age of enrolled patients was relatively high, the mean age was 71.59 ± 12.24 years old, and the TIR (80 to 190 mg/dL) corresponded to 54.73% and had a maximum Youden index. This cutoff value had good clinical significance.

The major advantage of our study lies in the utilization of the isCGM system for T2D patients complicated with COVID-19, enabling comprehensive assessment of hyperglycemia, hypoglycemia, and glycemic variability. Our study has presented the appropriate threshold and cutoff point for TIR in patients with COVID-19 and T2D, which is more relevant to clinical practice. However, several limitations need to be acknowledged. Firstly, it was a retrospective study, which may introduce patient selection bias. Secondly, the sample size was relatively modest and might not fully capture the complexity of the general population. Therefore, large-scale prospective cohort studies involving ethnically diverse cohorts from different geographical regions are warranted to gain a better understanding of the association between glycemic control and COVID-19 progression. Finally, it should be noted that our analysis excluded individuals with type 1 diabetes, but glycemic control could also influence their outcomes.

## 5 Conclusions

In conclusion, maintaining a TIR (80–190 mg/dL) above 54.73% independently correlates with a significant reduction in composite adverse outcomes associated with COVID-19 infection among patients with T2D. These findings provide valuable insights into the clinical characteristics of glycemic variability in individuals affected by both COVID-19 and T2D while offering potential avenues for improving disease outcomes.

## Data availability statement

The raw data supporting the conclusions of this article will be made available by the authors, without undue reservation.

## Ethics statement

The studies involving humans were approved by the Ethics Committees of Qilu Hospital of Shandong University. The studies were conducted in accordance with the local legislation and institutional requirements. The medical records or biological specimens used in this study are obtained from previous clinical treatment, and the risk to the subjects is not greater than the minimum risk, and the exemption from informed consent will not adversely affect the rights and health of the subjects. Subjects’ privacy and personally identifiable information will be protected by the researchers. The project leader solemnly promises not to use the medical records and specimens that patients have explicitly refused to use before. This research project does not involve personal privacy and commercial interests, and the samples and related information are only used for this research project.

## Author contributions

KT: Data curation, Formal analysis, Methodology, Supervision, Validation, Visualization, Writing – review & editing. RC: Methodology, Formal analysis, Writing – original draft. JBZ: Methodology, Formal analysis, Supervision, Writing – review & editing. LX: Formal analysis, Writing – original draft. YZ: Data curation, Writing – review & editing. HW: Data curation, Writing – original draft. JW: Data curation, Writing – original draft. WW: Data curation, Writing – original draft. YD: Data curation, Writing – original draft. HL: Data curation, Writing – original draft. JZ: Data curation, Writing – original draft.
